# Household satisfaction with a community-based health insurance scheme in Ethiopia

**DOI:** 10.1186/s13104-016-2226-9

**Published:** 2016-08-30

**Authors:** Abebe Sorsa Badacho, Kora Tushune, Yohannes Ejigu, Tezera Moshago Berheto

**Affiliations:** 1 School of Public Health, College of Health Sciences, Wolaita Sodo University, P.O. Box 138, Sodo, Ethiopia; 2 Department of Health Services Management, College of Public Health and Medicine, Jimma University, P.O. Box 378, Jimma, Ethiopia

**Keywords:** Community-based health insurance, Satisfaction score, Pilot district, Ethiopia

## Abstract

**Background:**

Community-based health insurance (CBHI) schemes are an emerging tool for providing financial protection against health-related poverty. In Ethiopia, CBHI is being piloted in 13 districts, but community experience and satisfaction with the scheme have yet to be studied. Therefore, this study aimed to assess the experiences and satisfaction of households enrolled in a pilot CBHI scheme.

**Methods:**

A community-based cross-sectional study method was used in one pilot district in South Ethiopia. Data were collected in March and April 2014. 386 households enrolled in the CBHI scheme were sampled by simple random sampling. Data were collected by trained data collectors using a pre-tested structured questionnaire. Descriptive statistics and bivariate and multiple linear regression analyses were performed. P values less than 0.05 and 95 % confidence intervals were used to determine associations between independent and dependent variables.

**Results:**

The study revealed that overall household satisfaction with CBHI was 91.38 %. Moreover, there was a significant association between health service provision and CBHI members’ satisfaction scores. For instance, household heads that strongly disagreed with laboratory services provision had an average 0.878 decrease in CBHI satisfaction score compared to household heads that strongly agreed. CBHI process- and management-related factors were also significantly associated with satisfaction.

**Conclusions:**

Satisfaction with CBHI was high. Age, family size, laboratory services provision, health services provider friendliness, CBHI offices opening times, membership card collection process, and time interval to use of services were significant predictors of satisfaction with CBHI.

## Background

Low-income countries face considerable challenges in financing healthcare [1–3]. Public services are unavailable and unaffordable to the majority of poor people in these countries [4]. Millions of people in developing countries still suffer and die from health-related conditions for which effective but underutilized interventions exist, particularly in settings that lack effective health insurance policies [5]. Health spending via out-of-pocket payments (OOPs) is difficult for many people, and 100 million people descend into poverty due to the need to pay for healthcare [6]. Health insurance can be a complementary or alternative source of healthcare finance [7] that has been implemented as part of health reform programs and strategies that aim to provide effective and efficient healthcare, in particular to the poor and vulnerable [8].

Several health insurance strategies can be used for pre-payment and universal coverage [9]. Most developed countries use tax-based systems, social health insurance systems, or mixed systems to achieve universal coverage [10]. Community-based health insurance (CBHI) schemes are an emerging and growing tool for providing financial protection to deprived individuals against health-related events. CBHIs have the following characteristics: voluntary membership, a non-profit objective, they are linked to a healthcare provider, they pool risk, and there is an underlying ethic of mutual aid trust, enrollment, and solidarity [11, 12].

Over 150 million people face catastrophic health expenditures each year, and most fall into poverty due to OOPs [13]; health problems and their associated costs are clearly an important cause of poverty, especially in countries that rely on OOPs [14]. In 60 % of countries with incomes below $1000 per capita, OOP spending constitutes over 40 % of total public healthcare expenditure [13]. About 1.3 billion people on very low incomes still lack access to effective and affordable drugs, surgeries, and other interventions due to weaknesses in health financing [15]. An absence of any form of health insurance increases the risk of poverty due to high healthcare-related costs. As the result, households may leave illness untreated or opt for the use of poor quality healthcare or self-administered medication [16]. Governments of low-income countries face the challenge of reducing the regressive burden of OOP expenditure by expanding pre-payment schemes that spread financial risk and reduce the spectrum of catastrophic healthcare expenditure [14].

Due to only limited access to a well-developed health insurance system, about 80 % of private health expenditure in Ethiopia is via OOPs and only 1.5 % of private healthcare expenditure is covered by private insurance institutions [17]. Providing healthcare to individuals working informally or who live in rural areas is a major challenge in developing countries [18, 19]. CBHI schemes are considered useful in addressing this problem. By pooling risks and resources, CBHIs promise better access to healthcare and risk protection for poor households against the cost of illness [20]. A national implementation pilot CBHI scheme was started in Ethiopia in mid-2011. As a starting point, thirteen districts were selected in four major regional states in Ethiopia for implementation of the pilot scheme. The aim was to reduce financial barriers and improve access to health services by reducing the burden of OOP expenditure [21]. The pilot program scheme covered both outpatient and inpatient healthcare services in public facilities with the aim of enhancing access to healthcare. The experiences and satisfaction of households enrolled in this pilot CBHI scheme is unknown. Therefore, this study focuses on the experiences and satisfaction of households in one of the pilot CBHI scheme districts in Ethiopia.

## Methods

### Study design

A community-based cross-sectional study was conducted in March and April 2014 in Damotwoyde district, 1 of the 13 districts selected for the CBHI pilot project. Damotwoyde district is located in the Wolaita zone in the Southern Nation Nationalities and Regional State of Ethiopia. The district has 23 rural kebeles (the lowest administrative unit in Ethiopia) and an estimated total population of 116,994. 50 % of the households in the district were enrolled in the CBHI pilot scheme. The district has four health centers and 25 health posts. The health coverage of the district was 92 % in 2014.

### Sample size determination

Sample size was calculated using Epi Info version 7 software using the single population proportion formula. Assuming 50 % of households enrolled in the CBHI scheme were satisfied with CBHI, a confidence level of 95 %, a 0.05 margin of error, and a 10 % non-response rate, the final sample size was 392.

### Sampling technique

All the districts selected for the pilot CBHI scheme were listed with the help of national CBHI agency officials. One of the 13 districts enrolled in the pilot CBHI scheme was selected by simple random sampling (Damotwoyde district). The total number of households enrolled in the pilot CBHI scheme in the selected district was identified using their individual enrolment identification numbers from registration book, and a total of 8008 households enrolled in the CBHI scheme; simple random sampling was used to obtain the final study participants registration book list as a frame.

### Data collection and quality assurance

A structured questionnaire was produced in English and translated from English to the local language. Another translator then translated the local version back into English to check for consistency of meaning. The study variables were adopted from the relevant literature.

The primary respondents were the household heads. Interviewer-administered face-to-face interviews were conducted using structured and pre-tested questionnaires. The data collectors were diploma holders who fluent in the local language. Data collectors were given 3-days training on the study objectives, method of data collection, and the tools for data collection. The supervisors were senior public health experts. The data collection tool was pre-tested in rural kebeles other than the study area; based on these results, adjustments were made to the data collection tool. Spot checks on the quality of data collection were made in the field and completed questionnaires were checked daily.

### Data analysis

Data were entered into EpiData version 3.1 and exported to SPSS version 20.0 for further analysis. The frequency distribution of all the variables was examined to check for data entry errors. Principal component extraction with eigen values greater than l and varimax rotation methods were employed for factor analysis. Items with Cronbach’s alpha values greater than 0.7 extracted from each of the scales were used in subsequent analyses. When the scales had more than one factor extracted, the factors were renamed according to the items contained in the extracted item. The item mean score and mean score of the scales were computed for those in Likert scale format. The factor score was computed for outcome variables and used for multiple linear regression. Variables showing a statistically significant association in bivariate analysis were analyzed using the enter method of multiple linear regression to examine associations between explanatory variables and the dependent variable. 95 % confidence intervals, and beta coefficients were calculated and used to describe statistically significant variables. A p value <0.05 was considered statistically significant.

### Measurements

#### Level of satisfaction: overall level of satisfaction with CBHI scheme

Six items related to satisfaction on a five point Likert scale (from 1: strongly disagree 1 to 5: strongly agree) were used to assess household heads’ satisfaction with CBHI. Together, all six items produced a maximum score of 30 and a minimum score of 6.

#### Health service-related factors

Household heads were asked four questions related to different aspects of service provision. Each question was scored on an ordinal scale from ‘strongly disagree’ to ‘strongly agree’ to yield a maximum score of 20 and a minimum score of 4. These four items were based on the following questions: satisfied with laboratory services; can get immediate care when visiting health facility; respect from service providers; services providers are friendly. The reliability coefficient (Cronbach’s alpha) of the health services-related factors scale was 0.851 indicating internal consistency.

#### CBHI process-related factors

Household heads were asked four questions on different aspects of CBHI process management on an ordinal scale from ‘strongly disagree’ to ‘strongly agree’ to yield a maximum score of 20 and a minimum score of 4. These four items were based on the following questions: I am satisfied with the opening hours of the CBHI office; I am satisfied with the collection process of insurance cards; I am satisfied with the time to make use of the CBHI program after payment of registration fee; and I am satisfied with the schedule for paying the premium. The reliability coefficient (Cronbach’s alpha) of the CBHI process management factors scale was 0.868 indicating internal consistency.

#### Outcome variables

The household heads’ overall satisfaction with the CBHI program was considered as an outcome variable. Six items related to satisfactions on a five point Likert scale were used to assess respondents’ satisfaction with CBHI to yield a maximum score of 30 and a minimum score of 6. These six items were based on the following questions: local CBHI management trustworthy; satisfied with information provided; satisfied with benefit packages; do not want to stay enrolled (inversely recoded) in the scheme; being enrolled in the scheme did not benefit the household because we are still spending on healthcare (inversely recoded); and recommending CBHI scale up to other settings (considered good if non-members become member of a CBHI scheme). Negatively stated questions were inversely recoded. To examine the underlying factors, an exploratory factor analysis was conducted, which produced one meaningful factor with an eigen value greater than one. This factor accounted for 60 % of the total variance and was renamed as the CBHI member’s satisfaction score; thus, the remaining scale items were discarded in linear regression.

*Ethical approval* Ethical approval was obtained from the ethical clearance board of Jimma University with reference number RPGC/445/2014. The participants were informed about the purpose of the study and oral consent `was obtained from each study participant prior to conducting the interview.

## Results

### Socio demographic characteristics of the respondents

Three hundred and eighty-six household heads participated in the study, producing a response rate of 98.4 %. Of these, 292 (75.6 %) were male. The median (interquartile range (IQR) age of participants was 44 (38.54) years. The majority of participants (274; 70.3 %) were married with one spouse. The median household size was 6, and 235 (60.9 %) had over five family members. Over half (206; 53.4 %) of participants were protestant. Nearly three-fifths and one in four participants were unable to read and write and attended primary school, respectively. 84.5 % (326) of participants were farmers. The estimated mean family income per annum as reported by respondent was 268.23 (SD ± 5.67) USD (Table 1).

**Table 1:** Socio-demographic characteristics of the participants, South Ethiopia, 2014 (n = 386)

Variables	Frequency	Percent
Sex of house hold heads		
Male	292	75.6
Female	94	24.4
Age		
25–44	191	49.4
45–64	147	38.1
>64	46	11.9
Marital status		
Married	299	77.4
Single	87	22.5
Family size		
1–5 members	151	39.1
Greater than 5	235	60.9
Religion		
Protestant	206	53.4
Orthodox	120	31.1
Catholic	60	15.5
Educational status		
Not able to read and write	236	61.1
Able to read and write	38	9.8
Grade 1–8	96	24.9
Grade 9 and above	16	4.1

### Experiences of participants in the pilot CBHI scheme

All participants reported that at least one family member had fallen sick and had visited a healthcare institution during their illness since enrolment in the CBHI scheme. About 80 % of household members who fell sick visited health centers within the district with a contractual agreement with the CBHI scheme; the remainder were referred to the nearest public hospital with an agreement with the CBHI scheme. Over 84 % of households visited healthcare institutions more than twice after scheme enrolment. Three quarters (290) of households reported that their decision to enroll as a member of the CBHI scheme was made by themselves. All respondents reported that they paid the membership fees.

Community-based health insurance members were required to visit public health centers within the district or the nearest public hospitals with an agreement with the CBHI scheme. Almost 98.2 % of household heads reported that they were happy with the permitted healthcare institutions. During their visits to healthcare institutions, 372 (96.4 %) of household heads received the correct prescribed drugs and 360 (93.3 %) reported that they received the requested laboratory services.

With respect to perceived service quality, 379 (98.2 %) participants perceived that the quality was good compared to their past experiences prior to CBHI. Similarly, 379 (98.2 %) participants reported that CBHI enrolment had benefited their household members. 304 (78.7 %) household heads had experience participating in a CBHI-related meeting.

#### Description of CBHI members’ satisfaction with the CBHI scheme

All household heads responded that they either agreed or strongly agreed in recommending CBHI scale-up to other settings. Almost all (97.8 %) respondents agreed or strongly agreed that scheme enrollment had benefited their households, while 376 (97 %) agreed or strongly agreed to stay enrolled in the scheme. Ninety-two percent of respondents responded that they either agreed or strongly agreed with local CBHI managers’ trustworthiness.

#### Level of satisfaction with the CBHI scheme

To determine the overall level of satisfaction with the CBHI scheme, internal consistency (Cronbach’s alpha) was first calculated for the scale items measuring satisfaction: the items had a Cronbach’s alpha of 0.861. The mean satisfaction was 27.93 ± 1.98 (possible range 6–30). The percentage mean (SD) satisfaction score was calculated based on the percentage of maximum scale score. Accordingly, the overall level of members’ satisfaction (percentage mean score) with CBHI was 91.38 ± 8.24.

#### Socio demographic predictors of members’ satisfaction with the CBHI scheme

Socio demographic variables explained only 7.7 % of satisfaction score variability. Accordingly, age, family size, and estimated annual income were statistically associated with satisfaction score. The CBHI members’ satisfaction score increased by an average of 0.011 units with age change in one year (95 % CIs 0.002 to 0.02). An average increase in family size decreased the satisfaction score by an average of −0.074 units (p < 0.05), with estimated annual income having no effect (Table 2).

**Table 2:** Socio-demographic determinants of CBHI members’ satisfaction of the CBHI scheme, South Ethiopia, 2014

Socio-demographic variables	No. (%)	p value	Unstandardized B coefficient	95 % CIs for B
Sex of household heads				
Male	292 (75.6)	0.867	−0.043	−0.541 to 0.456
Female^a^	94 (24.4)			
Age		0.021**	0.011	0.002 to 0.02
Marital status				
Married with one spouse^a^	274 (71)			
Married with more than 1	25 (6.5)	0.762	0.064	−0.351 to 0.480
Separated/divorced	13 (3.3)	0.175	−0.467	−1.144 to 0.209
Widowed	74 (19.2)	0.357	0.250	−0.283 to 0.78
Family size		0.008***	−0.074	−1.29 to −0.02
Religion				
Protestant^a^	206 (53.4)			
Orthodox	120 (31.1)	0.11	0.188	−0.043 to 0.418
Catholic	60 (15.5)	0.11	0.235	−0.053 to 0.524
Education				
Not able to read and write^a^	236 (61.1)			
Able to read and write	38 (9.8)	0.775	0.051	−0.299 to 0.041
Grade 1–8	96 (24.9)	0.157	0.180	−0.069 to 0.429
Grade 9 and above	15 (4.2)	0.71	0.097	−0.416 to 0.610
Household head’s occupation				
Farmer^a^	326 (84.5)			
Merchant	23 (6)	0.264	−0.252	−0.695 to 0.191
Housewife	37 (9.6)	0.304	0.238	0.693 to 0.217
Estimated annual income		0.003***	7.359	0.000 to 0.00

^a^References category (categories with highest frequency taken as reference categories)

*** Significant at p < 0.001, ** significant at p < 0.05, r^2^ = 7.7 %

#### Experiences of households since CBHI enrollment as a determinant of CBHI members’ satisfaction

Variables related to CBHI members’ experiences were entered into the model. This model explained 14.8 % of the variation in satisfaction among household heads within the CBHI scheme. Households who had paid the premium three times had an average decrease of 0.58 in CBHI satisfaction compared to households who paid the premium over three times (p < 0.05). Households who paid the premium twice a year had an average decrease of 0.32 in CBHI satisfaction compared to households that paid monthly (p < 0.05; Table 3).

**Table 3:** Different experiences of CBHI as determinants of CBHI members’ satisfaction with the scheme, South Ethiopia, 2014

Different experiences	No. (%)	p value	UnstB coefficient	95 % CIs for B
Voluntary enrolment in the CBHI				
Yes^a^	310			
No	76	0.036^a^	−0.298	−0.58 to −0.02
Got prescribed drugs				
Yes^a^	372			
No	14	0.563	−0.173	−0.76 to 0.41
Got requested laboratory services				
Yes^a^	360			
No	26	0.290	−0.260	−0.74 to 0.22
Satisfied with visited healthcare institution				
Yes^a^	375			
No	11	0.667	−0.174	−0.97 to 0.62
CBHI benefited households				
Yes^a^	379			
No	7	0.003**	−1.25	−2.00 to −0.42
Participation of CBHI-related meeting				
Yes^a^	304			
No	82	0.49	−0.33	−1.30 to 0.64
Discussion with CBHI managers				
Yes^a^	300			
No	86	0.89	0.07	−0.90 to 1.00
Times premium paid				
Once	10	0.61	−0.156	−0.76 to 0.45
Twice	37	0.95	0.011	−0.32 to 0.35
3 times	64	0.001**	−0.578	−0.84 to −0.32
>3 times^a^	275			
Times healthcare visited				
Once	59	0.36	0.14	−0.16 to 0.43
Twice	98	0.98	0.003	−0.25 to 0.25
3 times	91	0.53	0.08	−0.18 to 0.34
>3 times^a^	138			
Schedule of payment				
Monthly^a^	255			
Quarterly	60	0.154	−0.202	−0.48 to 0.07
Twice per year	62	0.024^a^	−0.320	−0.59 to −0.04
Once a year	9	0.503	−0.218	−086 to 0.422

^a^Reference group

*** significant at p < 0.001, ** Significant at p < 0.05

#### Health service provision-related determinants of CBHI members’ satisfaction with the CBHI scheme

Variables related to health service provision were entered into the model. This model explained 19.2 % of the variation in satisfaction among household heads in the CBHI scheme. Respondents who strongly disagreed that they were satisfied with laboratory services provision had a 0.88 average decrease in CBHI satisfaction score compared to household heads who were strongly agreed (p < 0.001). Furthermore, household heads who strongly disagreed with service providers’ friendliness had an average decrease of 0.82 in the CBHI satisfaction score compared to household heads who were strongly agreed (p < 0.001; Table 4).

**Table 4:** Health services provision-related determinants of CBHI members’ satisfaction of CBHI scheme, South Ethiopia, 2014

Variables	No. (%)	P value	Unstandardized B coefficient	95 % CIs for B
Satisfied with laboratory services				
Strongly disagree	17 (4.4)	0.000**	−0.88	−1.34 to −0.42
Disagree	17 (4.4)	0.012**	−0.59	−1.06 to −0.13
Neutral	6 (1.6)	0.07	−0.67	−1.423 to 0.08
Agree	163 (42.20)	0.07	−0.19	−0.39 to 0.02
Strongly agree^a^	183 (47.4)			
Services providers friendly				
Strongly disagree	43 (11.1)	0.000***	−0.82	−1.13 to −0.51
Disagree	37 (9.6)	0.000***	−0.78	−1.10 to −0.45
Neutral	9 (2.3)	0.000***	−1.16	−1.77 to −0.54
Agree	95 (24.6)	0.000***	−0.33	−0.57 to −0.09
Strongly agree^a^	202 (52.3)			

^a^Reference category

*** Significant at p < 0.001, ** significant at p < 0.05, r^2^ = 19.2 %

#### CBHI process and management-related determinants of CBHI members’ satisfaction

Variables related to CBHI process and management were entered into the model. The model explained 32.6 % of the variation in satisfaction in household heads enrolled in the CBHI scheme. The card collection process, CBHI office opening times, time interval to use benefits, and paying the premium were all significantly associated with member satisfaction (Table 5).

**Table 5:** CBHI process and management-related determinants of satisfaction with CBHI scheme, South Ethiopia, 2014

Variables	No (%)	P value	Unstandardized B coefficient	95 % CI for B
Happy with CBHI offices opening times				
Strongly disagree	4 (1)	0.606	0.220	−0.62 to −1.06
Disagree	6 (1.6)	0.000***	−1.578	−2.311 to −0.844
Neutral	3 (.8)	0.789	−0.225	−1.883 to −1.433
Agree	157 (40.7)	0.005***	−0.258	−0.44 to −0.077
Strongly agree^a^	216 (56)			
Satisfaction with members card collection on process				
Disagree	4 (1)	0.447	0.357	−0.565 to 1.280
Neutral	3 (.8)	0.063	1.200	−0.066 to 2.466
Agree	119 (30.8)	0.000***	−0.520	−0.723 to −0.318
Strongly agree^a^	260 (67.4)			
Satisfied with time interval to use benefit package				
Disagree	14 (3.7)	0.053	−0.506	−1.018 to 0.006
Neutral	2 (0.5)	0.004***	−3.281	−5.488 to −1.073
Agree	91 (23.6)	0.567	−0.066	−0.294 to 0.161
Strongly agree^a^	279 (72.3)			
Satisfied with paying premium				
Strongly disagree	1 (.3)	0.239	−0.999	−2.664 to 0.667
Disagree	8 (2.1)	0.379	−0.288	−0.929 to 0.354
Neutral	4 (1)	0.000***	−2.705	−3.758 to −1.653
Agree	102 (26.4)	0.006***	−0.295	−0.506 to −0.084
Strongly agree^a^	271 (70.2)			

^a^Reference category

*** Significant at p < 0.001, ** significant at p < 0.05

Household heads who disagreed with CBHI office opening times had an average 1.578 CBHI satisfaction score decrease compared to household heads that were strongly agreed (p < 0.001), while household heads who agreed had an average decrease of 0.258 in the satisfaction score compared to those who strongly agreed (p < 0.01). Two hundred and sixty (67.4 %) household heads responded that they strongly agreed with the card collection process. Household heads that agreed had an average decrease of 0.52 in satisfaction score compared to those who strongly agreed (p < 0.01). Household heads who were neutral for satisfaction with the time taken after registration and use of the services or waiting time as a mechanism to reduce or control adverse selection of CBHI members had an average decrease of 3.281 compared to household heads who were strongly agreed (p < 0.01). Three hundred and seventy-three (96.6 %) household heads responded that they strongly agreed or agreed with paying membership premium and those that agreed had a 0.295 decrease in satisfaction score compared to those who strongly agreed (p < 0.01).

#### Predictors of CBHI members’ satisfaction with CBHI

Multivariable regression was performed for significant variables in bivariate analysis to produce a final model for CBHI scheme satisfaction. Accordingly, age, time premium paid, laboratory services provision, health services provider friendliness, CBHI office opening time, card collection process, time interval, and use of services (waiting period to control adverse selection of members) were found to be strong predictors.

On average, an increase in family size produced an average decrease of 0.047 in their satisfaction score (95 % CIs −0.09 to −0.005), while 1-year increase in household head age increased satisfaction by0.09 (p < 0.05). Household heads that strongly disagreed with laboratory services provision had an average decrease of 0.13 units in satisfaction compared to household heads who agreed (p < 0.05). Household heads who strongly disagreed with service provider friendliness had an average decrease of 0.12 units in satisfaction (p < 0.05), while those that disagreed with local CBHI scheme office opening times had an average decrease of 0.16 units in satisfaction compared to those who strongly agreed (p < 0.001). Similarly, household heads that agreed with the membership card collection process had a decrease of 0.18 units in satisfaction score compared to those who strongly agreed (p < 0.001; Table 6).

**Table 6:** Predictors of CBHI members’ satisfaction with CBHI, Damot woyde, Southern Ethiopia April 2014

Variables	No (%)	P value	Unst B	Stan B	95 % CI for B
Age		0.03**	0.007	0.09	0.001 to 0.015
Family size		0.055	−0.04	−0.09	−0.08 to 0.001
Income		0.259	0.00	0.05	0.000 to 0.000
Lab services					
Strong disagree	17 (4.4)	0.009***	−0.58	−0.12	−1.01 to −0.15
Disagree	17 (4.4)		0.02	0.05	−0.40 to 0.45
Strongly agree^a^	183 (47.4)	0.914			
Services provider friendly					
Strongly disagree	43 (11.1)		−0.420	−0.13	−0.75 to −0.09
Disagree	37 (9.6)	0.012**	−0.359	−0.11	−0.68 to −0.04
Neutral	9 (2.3)	0.03**	−0.903	−0.136	−1.45 to−0.35
Agree	95 (24.6)	0.001**	−0.18	−0.08	−0.38 to 0.03
Strongly agree^a^	202 (52.3)	0.09			
Happy with CBHI offices working time					
Disagree	6 (1.6)	0.001***	−1.311	−0.16	−2.09 to −0.53
Agree	157 (40.7)	0.004**	−0.254	−0.12	−0.42 to −0.08
Strongly agree^a^	216 (56.0)				
Card collection process					
Agree	119 (30.8)	0.001***	−0.4	−0.18	−0.59 to −0.21
Strongly agree^a^	260 (67.4)				
Time interval and use					
Neutral	2 (.5)	0.001***	−3.12	−0.22	−4.47 to −1.76
Strongly agree^a^	279 (72.3)				
Pay premium					
Agree	102 (26.4)	0.001***	−1.83	−0.19	−2.71 to −0.95
Neutral	4 (1)	0.04**	−0.31	−0.12	−0.54 to −0.09
Strongly agree^a^	271 (70.2)				
Voluntary enrollment					
Yes^a^	310				
No	76	0.121	−0.19	−0.07	−0.43 to −0.05
CBHI benefited households					
Yes^a^	379				
No	7	0.99	0.002	0.00	−0.7 to 0.70
Schedule of payment					
Monthly^a^	255				
Twice per year	65	0.216	−0.15	−0.05	−0.38 to 0.08
Times premium paid					
3 times	64	0.007**	−0.31	−0.12	−0.54 to −0.08
>3 times^a^	275				

^a^Reference category

*** Significant at p < 0.001, ** significant at p < 0

## Discussion

Establishing health insurance services is gaining traction in resource-limited countries to improve health care utilization and ensure financial protection for households to mitigate against poverty induced by OOPs. Despite this, the impact of health insurance in low and middle-income countries is, unfortunately, poorly documented.

**Fig. 1:**
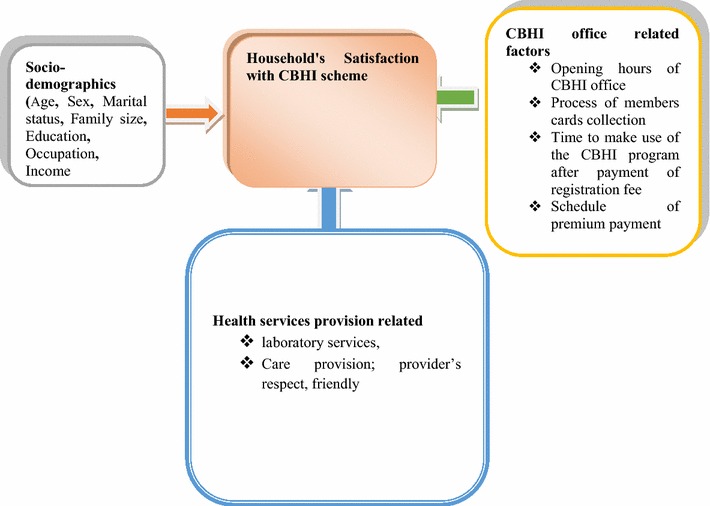
Conceptual framework for household satisfaction with a community-based health insurance scheme in Ethiopia developed from [3, 13, 16, 17, 19, 22]

Here we show that household satisfaction with a CBHI scheme implemented in Ethiopia was high (Fig. 1). In 2005, the World Health Assembly called on all countries to move towards universal health coverage, especially in developing countries with huge inequalities in health service delivery [22, 23]. Thus, the overall high satisfaction rate shown here may help and encourage scale up of the CBHI scheme to the remainder of the community and enhance universal health coverage (UHC). There is evidence that CBHI increases healthcare utilization [12]. In this study, age was associated with CBHI satisfaction; in a similar study in Nigeria, older clients were more satisfied with service provision than younger clients [24]. This might be attributed to more frequent illness in elderly people. However, other variables such as occupation, education, marital status, sex, and income were not significant predictors, consistent with studies conducted in India on national health insurance satisfaction, who also found no differences in satisfaction across socioeconomic and demographic variables [3]. Conversely, a Turkish study of patient satisfaction of national health insurance showed that there was a significant relationship between satisfaction and gender, marital status, education level, and occupation [25]. This may be due to differences in study design, since the Turkish study was facility based. We also found that an average increase in family size reduced satisfaction with the CBHI scheme. This may have been due to payment arrangements, since larger families incur additional fees, which must be declared as the family grows in size.

We also aimed to identify health service provision-related factors that were significantly associated with CBHI satisfaction; good laboratory service provision and health provider friendliness during service provision increased satisfaction with CBHI. This suggests that, and bearing in mind a “chicken or egg” dilemma as a limitation of the study, that satisfaction with involvement in CBHI increased expectation/satisfaction in laboratory services and provider friendliness. In a similar study in Burkina Faso, CBHI members’ poor perception of their healthcare provider was an important reason for dropping out of the Nouna Community Based Insurance scheme [26]. Another study from rural tropical Ecuador found that low healthcare utilization could be an obstacle to successful implementation of a CBHI scheme and was closely associated with the local health services and availability of dedicated and friendly staff and essential drugs [1]. Furthermore, a study on India’s national health insurance scheme implied that the services provided by doctors and nurses were slightly less satisfactory [3].

We also found that CBHI process and management were significantly associated with CBHI satisfaction, particularly with respect to CBHI office opening times, the membership card collection process, waiting time (length of time between registration and use of the service), and amount of payment, which were all positively associated with satisfaction. In a study of Kenya’s Kilifi district CBHI scheme, households reported that they were not interested in renewing their membership due to corruption affecting CBHI management and leading to dissatisfaction [27].

Finally, our findings indicate that age, family, laboratory services, service provider friendliness, office opening times, card collection process, time interval to use of services (waiting period), paying the premium, and the number of times the premium was paid were the main predictors of CBHI members’ satisfaction with this CBHI scheme.

## Study limitation

This study only focused on the demand side point of view (households who enrolled), and the supply side view (provider side) was not explored.

## Conclusions

Based on these findings, households enrolled in CBHI are highly satisfied and almost all households would recommend expansion of the program to other settings. Rural and informal sectors in particular benefited from this program. Due to their enrollment in the CBHI pilot program, members were able to access healthcare and utilize modern healthcare institutions. Age, family size, frequency of premium paid, laboratory services provision, friendliness of health services providers, CBHI office opening times, the membership card collection process, and time interval to use of services were significant predictors of satisfaction with the CBHI scheme.

## Recommendation

To achieve universal health coverage, the Government of Ethiopia is promoting and implementing voluntary CBHI schemes and social health insurance to enhancing healthcare access and reduce the burden of OOP expenditure. This was a study of a pilot CBHI scheme, with promising and encouraging results for rural households. However, we recommend further studies to identify other predictors of CBHI members’ satisfaction not addressed here, especially health service provision-related determinants.
